# Rational Use of Medicines in Neonates: Current Observations, Areas for Research and Perspectives

**DOI:** 10.3390/healthcare6030115

**Published:** 2018-09-14

**Authors:** Karel Allegaert

**Affiliations:** 1 Department of Pediatrics, Division of Neonatology, Erasmus MC-Sophia Children’s Hospital, Doctor Molenwaterplein 40, 3015 GD Rotterdam, The Netherlands; k.allegaert@erasmusmc.nl or karel.allegaert@uzleuven.be; 2 Department of Development and Regeneration, KU Leuven, Herestraat 49, 3000 Leuven, Belgium

**Keywords:** newborn, rational drug utilization, perinatal pharmacology, clinical pharmacology, effective implementation

## Abstract

A focused reflection on rational medicines use in neonates is valuable and relevant, because indicators to assess rational medicines use are difficult to apply to neonates. Polypharmacy and exposure to antibiotics are common, while dosing regimens or clinical guidelines are only rarely supported by robust evidence in neonates. This is at least in part due to the extensive variability in pharmacokinetics and subsequent effects of medicines in neonates. Medicines utilization research informs us on trends, on between unit variability and on the impact of guideline implementation. We illustrate these aspects using data on drugs for gastroesophageal reflux, analgesics or anti-epileptic drugs. Areas for additional research are drug-related exposure during breastfeeding (exposure prediction) and how to assess safety (tools to assess seriousness, causality, and severity tailored to neonates) since both efficacy and safety determine rational drug use. To further improve rational medicines use, we need more data and tools to assess efficacy and safety in neonates. Moreover, we should facilitate access to such data, and explore strategies for effective implementation. This is because prescription practices are not only rational decisions, but also have psychosocial aspects that may guide clinicians to irrational practices, in part influenced by the psychosocial characteristics of this population.

## 1. Introduction

*“Why a focused reflection on rational use of medicines in neonates is valuable and relevant.”*

The World Health Organization (WHO) defines the rational use of medicines as the use of medicines so that individual patients receive medicines that are appropriate to their clinical needs, in doses in accordance with their own individual requirements, for the appropriate period of time, and at the lowest or reasonable cost to both the individual and the community [1]. Rational use of medicines relates to: (i) an *accurate strategy and monitoring on medicines use* (advocating rational medicines use, identifying and promoting successful strategies, and securing responsible medicines promotion); (ii) *rational use of medicines by health professionals* (develop and update national guidelines, essential medicines list, and training on rational medicines use); and (iii) *rational use of medicines by consumers/patients* (develop and support effective systems of medicines information and empower consumers/patients to take responsible and well-educated decisions regarding medicines use) [1].

The WHO estimate that more than 50% of the medicines are prescribed, dispensed or sold inappropriately and that about 50% of patients fail to take their medicines correctly. These inaccuracies result in wastage of resources, additional health risks, suboptimal management or failure to use proven more effective interventions (failed opportunity cost). Commonly used indicators of potential irrational practices are the incidence of polypharmacy, inappropriate use of antibiotics (dose, indication, duration, and route of administration), inappropriate self-medication, or failure to adhere to clinical guidelines or dosing regimens [1]. Consequently, irrational use of medicines is a major and global problem, but has specific issues when we focus on rational medicines use in neonates [1,2,3]. These issues relate to current practices and areas in need of additional research.

As documented in the latest Pediatrix analysis (2005–2010, *n* = 450,386 neonates), only 35% of the medicines were FDA-approved. Polypharmacy in neonates turned out to be common, with a mean number of medicines courses of 4 (1–14) per infant, raising up to 17 (2–45) courses in extreme low birth weight (ELBW < 1 kg) infants. Moreover, 16 antibiotics were observed in the Top 50, and 3 (ampicillin, gentamicin, and vancomycin) in the Top 5 most commonly administered drugs [4]. The majority of drugs in neonates are prescribed and administered in a hospital setting. Consequently, inaccurate exposure to medicines almost exclusively relates to organizational issues and drug errors within health care facilities. Dosing errors were identified as the most common error, with computerized physician order entry and interventions by clinical pharmacists as most common interventions suggested to reduce drug errors in neonates [2,5]. The risk for errors in neonates is further increased because of the absence of sufficient evidence on pharmacotherapy and the lack of formulations tailored to neonates [2].

Compared to the available data on benefits and risks to make rational decisions on medicine use in adults, the existing information to make such informed decisions in neonates is much more limited. Off-label or unlicensed use of drugs remains most prevalent in neonates [6]. Medicines utilization research serves as a tool to assess if medicines use is rational. In neonates, this goal is largely hampered because of the extensive off-label and unlicensed medicines use. *Quality* (compare practices to guidelines and local drug formularies), *patterns* (extent or profiles of drug use and trends), *outcomes* (health outcomes and both benefits and adverse effects) or *determinants* (prescriber characteristics and impact of interventions) can still be explored as potential indicators of irrational practices [7,8].

In this narrative review on rational medicines use in neonates, we highlight trends on anti-reflux drugs (Section 2.1), analgesics (Section 2.2) and anti-epileptics (Section 2.3) as Anatomical Therapeutic Chemical (ATC) Classification System classes to illustrate issues related to implementation of rational medicine use in this population. This is preceded by a short topical introduction on the specific characteristics of neonatal clinical pharmacology, and followed by highlighting some areas for future research (breastfeeding (Section 3.1) and drug safety assessment in neonates (Section 3.2)). We deliberately do not cover aspects related to antibiotics, since patterns of empiric antibiotic administration, the extensive variability in practices, the limited evidence to support practices, and strategies to implement antimicrobial stewardship have recently been described [9,10,11,12,13,14].

## 2. Neonatal Clinical Pharmacology

*“Extensive variability in dosing and effects are the key characteristics of neonatal clinical pharmacology. The impact of medicines to improve outcome and rational medicines use in neonates remains underexplored.”*

When clinicians prescribe medicines to neonates, it is with the intention to provide the newborn an effective intervention, avoiding disproportional side-effects. Clinical pharmacology aims to estimate the effects of such interventions, using pharmacokinetics (PK) and pharmacodynamics (PD) to generate predictions, including a grade of certainty and confidence intervals. PK (absorption, distribution and elimination, through either metabolism or renal elimination) estimates the concentration–time relationship. PD aims to estimate (side-)effects of a specific medicine (concentration–effect relationship). Very specific to newborns are the fast maturational changes in neonatal life with weight and age as main drivers of this maturation, resulting in extensive between- and within-individual variability in PK and subsequent PD [15]. Non-maturational changes further add to the variability. Observed non-maturational changes relate to co-medication such as ibuprofen, pharmacogenetics or co-morbidity (sepsis snf asphyxia) [16]. At best, this means that dosing regimens of medicines in newborns should be based on integrated knowledge concerning the specific diseases to be treated, the maturational and non-maturational characteristics of the newborn, and the PK and PD estimates of the medicine.

Consequently, the potential health impact of neonatal pharmacotherapy remains underexplored. It is still very common practice to administer medicines outside their authorization. Although off-label is not necessarily equal to off-knowledge, this does result in the fact that clinicians commonly lack the crucial information to make the best possible, informed decision. Unfortunately, history repeatedly provided evidence that newborns are more prone to specific adverse reactions to drugs, although some of these adverse reactions may have been anticipated based on the available knowledge on developmental pharmacology illustrated in Table 1 [2,3].

In the current narrative review, we focus on drug utilization of specific ATC groups, such as drugs for peptic ulcer and gastroesophageal reflux (Section 2.1), analgesics (Section 2.2) or anti-epileptics (Section 2.3), in neonates; drug utilization studies to illustrate how such studies can inform us on trends over time; between unit variability; and the impact of implementation of guidelines on drug utilization patterns.

### 2.1. Drugs for Peptic Ulcer and Gastroesophageal Reflux Disease (ATC Class A02B)

*“We should be aware that a shift in practices because of a documented safety issue may result in a subsequent irrational practice with another drug.”*

Gastroesophageal reflux is commonly defined as the passage of acid or non-acid gastric fluids back into the esophagus. This diagnosis is frequently made during neonatal stay, and is claimed to be link to apnea-bradycardia events [17]. While cisapride decreases the incidence of GOR events, the impact on reflux-related apnea was much weaker [18]. Once this compound was withdrawn from the market because of cardiac arrhythmia events, there was a shift to acid reducing drugs and other pro-kinetics. This occurred despite the lack of evidence that gastric acid reducing drugs are effective to reduce gastric acid production, reflux and apnea. More recently and following this shift, there was also emerging evidence on relevant harm (higher incidence of necrotizing enterocolitis or late onset sepsis) [17,19].

Using the analyses on trends as published in two consecutive Pediatrix (1997–2004 and 2005–2010) cohorts, the impact of these findings into clinical practices are reflected [4,20]. In the first (1997–2004) cohort, there was a significant decrease for cisapride (7.5% to 0%, neonates exposed during neonatal stay). However, this decrease was mirrored by a significant increase in metoclopramide (2.2% to 10%) and ranitidine (5.5% to 8%) between 1997 and 2004, while proton pump inhibitors (PPIs, omeprazole and pantoprazole) were not yet present in the Top 30 list of most commonly administered drugs in neonates. In the second (2005–2010) time interval, Hsieh et al. observed a subsequent decrease in metoclopramide (−84%, from 88 to 14/1000 neonates) and ranitidine (−61%, from 80 to 31/1000 neonates), mirrored by an increase in lanzoprazole (+58%, from 9.8 to 16/1000 neonates) exposure. The stable prescription of erythromycin (27th and 24th most commonly prescribed drug in the consecutive cohorts) also illustrates the limitations of such analysis without simultaneous collection of data on indications (infection related or as prokinetic) [4,20]. A strategy how to implement a guideline (set at target, plan–do–study–act, feedback and education) to decrease the use of acid-suppressing medicines in a neonatal intensive care unit has recently been described [21].

### 2.2. Analgesics (ATC Class N02)

*“Increased exposure to analgesics and the extensive variability observed in drug prescription practices is concerning given the limited evidence of benefit and potential harm. Implementation strategies to structure rational use of analgesics are effective.”*

Effective analgesia in neonates is relevant not only because of ethics or empathy, but also since a crucial and valid part of contemporary nursing and medical practice. However, and resulting in the need for a balanced approach, there is also emerging evidence on the extent of exposure to analgesics and poorer neurodevelopmental outcome in neonates. Consequently, the increased exposure to analgesics over time and the extensive variability observed in drug prescription practice is concerning given the limited evidence of benefit and the potential harm [4,20,22,23,24].

The Pediatrix consortium reported on medication use in two consecutive time intervals (1997–2004 and 2005–2010). When we compare these cohorts, fentanyl and morphine were in the Top 30 list (19th and 25th) with an estimated exposure of 56 and 35/1000 admitted neonates in the 1997–2004 cohort, respectively, and were observed seventh and 14th (estimated exposure of 70 and 51/1000 neonates, respectively) in the 2005–2010 cohort [4,20,24]. Using the same Pediatrix dataset (1997–2012) with focus on the use of analgesics, sedatives and paralytics in ventilated preterms (<1500 g, <32 weeks, *n* = 8591), Zimmerman et al. documented that—despite the meta-analytical evidence [25]—opioid exposure increased from 5% to 32% of ventilation days [24]. In the Canadian network, extensive between unit variability in prescriptions was observed (3–41% analgesics), not explained by clinical characteristics [23]. A similar pattern (common (26%) opioid exposure and extensive variability) has been reported in the EUROPAIN cohort [22].

Implementation exercises of guidelines on opioid use are effective to reduce the utilization of these drugs and its variability [26]. This reduction in exposure was reflected in the number of patients (from 63% to 33%) and the cumulative morphine dose (from 1.64 to 0.51 mg/kg, −68%). Interestingly, this implementation effort also resulted in a significant reduction in the number of cases (from 10/205 to 3/250 cases, *p* < 0.05) requiring methadone treatment for iatrogenic NAS syndrome [26].

### 2.3. Anti-Epileptic Drugs (ATC Class N03A)

*“Rational use of AED medicines necessitates the development of more advanced tools to improve the accuracy of the diagnosis, while better knowledge on pathways involved in the ‘seizures’ phenotype in neonates is needed to further individualize treatment.”*

The Pediatrix consortium reported on the prescription of anti-epileptic drugs (AED) in two consecutive time periods (1997–2004 compared to 2005–2010), analyzing the same administrative database. Phenobarbital was prescribed in 4.5% and 3.8% of cases in these time intervals, and midazolam in 3.8% and 6.1%. [4,20]. More recently, but using the same Pediatrix database (2005–2014, *n* = 9134/778,395, 1.1% with seizures), Ahmad et al. confirmed that neonates with seizures are still overwhelmingly exposed to phenobarbital with a very minor decease over the studied time interval (from 99% to 96%), a decrease in phenytoin use (from 15% to 11%), and a very relevant increase in levetiracetam (from 1.4% to 14%) with carbamazepine, lidocaine or topiramate as rarely administered AEDs in neonates (all < 1%) [27]. Similar to the earlier mentioned issue on erythromycin use, it is also difficult to assess trends in the prescription of benzodiazepines when either seizures or sedation is aimed for. Despite this comment, we can still reflect about the available data.

First, the still overwhelming use of phenobarbital as first line AED is suboptimal and unique to neonates, since cumulative phenobarbital exposure is associated with a decrease in cognitive and motor Bayley scores (8 and 9 IQ points, respectively, for every 100 mg phenobarbital/kg body weight), and a higher risk for cerebral palsy (2.3-fold) [28]. Second, different approaches have been considered to proceed to better evidence and rational drug utilization. These strategies included more advanced technology with bedside neuro-physiological monitoring (*reduce the number of inaccurate diagnoses*) instead of clinical observations based on motor or autonomic events, and strategies to reduce the mean phenobarbital burden and reduction in the incidence of AED exposure at discharge (*reduce the duration of exposure in treated newborns*) [29]. Even more promising, better insights into the variety of mechanisms (asphyxia, infarction, channelopathies, and metabolic syndromes) involved in the “seizure phenotype” should enable us to shift from a “one drug fits all” approach to individualized pharmacotherapy (*better mechanism driven medicine selection*) [30].

## 3. Areas in Need of Research on Perinatal Drug Exposure

### 3.1. Breastfeeding

*“It is a misconception that a ‘when in doubt, do not provide breastfeeding’ approach has no negative effects. We need new tools to quantify breastfeeding related exposure and tools to provide access to this information.”*

Lactating women regularly take medicines, and are commonly advised to interrupt or even stop nursing while taking medicines, even though only a limited number of medicines have been identified as (likely) harmful. Since breastfeeding itself also provides clinically relevant benefits to both mother and infant, it is a misconception that “*when in doubt, do not provide breastfeeding*” has no negative effects [31,32]. Consequently, the overarching intention of maternal intake of medicines during nursing should fulfill two criteria: (i) provide effective and safe medicines for the mother; and (ii) assure safety of nursing newborns from adverse events related to these medicines following breastfeeding related exposure as well as the adverse events related to the interruption of breastfeeding [31,32]. The relevance of these balanced decisions is illustrated with the impact of breastfeeding on neonatal abstinence syndrome (NAS) and the neurodevelopment outcome following breastfeeding related exposure to anti-epileptic drugs (AED) following fetal exposure.

The effects of breastfeeding on the incidence and severity of opioid-related NAS have been quantified. In essence, there is a significant reduction in the incidence (number needed to treat 5–6) and the severity of NAS during breastfeeding [33]. Similarly, the impact of breastfeeding on neurocognitive outcome following AED exposure during pregnancy and lactation has been documented by the Neurodevelopmental Effects of antiepileptic drugs (NEAD) group. At six years, children of mothers on AEDs had a higher (11.5% instead of 4.8%) risk of impaired fine motor skills compared with controls and a lower and dose dependent IQ (from −6 to −9 IQ point) following fetal valproate exposure when compared to other AEDs. Building on these background characteristics, subsequent breastfeeding in infants of women using AEDs was associated with *improved* neurodevelopment outcome compared with those with either no breastfeeding or breastfeeding for less than six months [34,35]. Overall, the adjusted IQ was higher by four points for children who were breastfed compared to those who were not. The difference in IQ was most pronounced in the valproate group (+12, range, 1–24 IQ) [35]. To facilitate more rational decisions on this topic, we need more advanced tools to generate knowledge, and we should subsequently ensure access to this evolving knowledge.

Physiologically-based pharmacokinetic (PB and PK) modeling tools are evolving and have been used to assess fetal or infant exposure to toxic compounds such as perfluoro-alkyl substances or mercury [36]. These models can also be applied to convert observations on drug levels in human milk into a population distribution to assess drug exposure and subsequent safety in newborns. Using a rather small dataset of 18 breastfeeding women on escitalopram, PB and PK methods served as tool to generate an average estimate (1.7%, range 0.5–5.9%) of the maternal plasma area under the curve [37]. However, such approaches mainly estimate population average exposure, not necessary covering all individual outlier patterns due to, e.g., polymorphisms [38].

There are different initiatives to subsequently ensure access to these data. LactMed is a free, easily accessible online database with information on drugs and lactation as one of the newest additions to the National Library of Medicine’s TOXNET system [39]. Such an online tool facilitates updates when additional information becomes available. Along the same line, and related to the Pregnancy and Lactation Labeling Final Rule, the FDA requests a more narrative description of the available evidence on medicines use during pregnancy in three subsections, including “lactation”. The “lactation” section hereby aims to provide information about the use of medicines while breastfeeding, such as the amount of medicines observed in human milk and effects on the nursing infant [40].

### 3.2. How to Assess Drug Safety in Neonates

*“How to retrieve, qualify and quantify the drug safety signal in the noise.”*

An adverse event is any untoward medical occurrence in a patient or trial participant exposed to a medicine. Adverse event assessment relates to seriousness, causality and severity, but all have their issues when applied to neonates. *Seriousness* is a regulatory concept, but prolongation of existing hospitalization in a (preterm) newborn is sometimes difficult to assess. *Causality* necessitates the disentangling of adverse drug events from confounding events such as organ dysfunction, maturational changes or co-morbidity. The more commonly used tools such as the Naranjo algorithm do not sufficiently reliably document causality in neonates, and none of these tools has been validated in this setting. A specific tool to assess causality in neonates has been suggested, but has not yet undergone prospective validation in the clinical setting [41]. The same holds true for *severity* (mild, moderate, severe, life threating or death) grading. Severity scales exist, but none of these grading scores is fully tailored to neonates. The generic criteria (instrumental activities of daily life (ADL), or self-care) are not applicable to neonates, and typical neonatal adverse events are still lacking. This necessitates development in a consensus approach, with subsequent prospective validation. At least, harmonization of the adverse event assessment enables clinicians, parents and regulatory bodies to compare treatment modalities on their effects and side-effects.

## 4. Discussion: Perspectives to Further Improve Rational Use of Medicines in Neonates

*“We like to believe that decisions on medicines use in neonates are driven by rational processes, but we should also explore the psychosocial aspects that guide our decisions.”*

Using a narrative approach, we illustrate that a focused reflection on rational medicines use in neonates is valuable and relevant. We hereby documente that polypharmacy and exposure to antibiotics are very common, while dosing regimens or clinical guidelines are only rarely supported by robust evidence in neonates. Consequently, extensive variability in practices is observed. Medicines utilization research informs us on trends, on between unit variability and on the impact of guideline implementation. This utilization research is not limited to exposure to antibiotics [4,9,10,11,12,13,14,20], but has been illustrated using data on drugs for gastroesophageal reflux, analgesics or anti-epileptic drugs [4,17,18,19,20,21]. We hereby illustrate how a shift in practices can result in another irrational practice with another compound (shift from prokinetics to anti-acid drugs to treat reflux associated-apnea). Extensive variability in the use of analgesics is observed. For both ATC groups, research on implementation strategies proved to be effective [21,26]. For AEDs, we use the data on the minor shifts in drug utilization patterns to illustrate the relevance to develop tools for more accurate diagnosis, and guidelines to reduce the duration of exposure [27]. Finally, we need more data on the mechanisms involved in the “neonatal seizures phenotype” to shift away from the currently “one drug fits all” approach to a more individualized AED use [30].

The need to develop more tailored tools to assess exposure, efficacy and safety of medicines in neonates is not limited to these ATCs or clinical syndromes. This was subsequently illustrated by the need to generate additional tools to predict medicine related exposure through breastfeeding and to facilitate access to data (online tools and regulatory initiatives). Similarly, tailored tools are needed to assess all aspects of safety (seriousness, causality, and severity tailored to neonates) since both efficacy and safety determine rational medicine use: *How can the signal in the noise be recognized?*

However, we should be aware that clinical decision making and drug utilization practices are not only just rational decisions, but also have psychosocial aspects that may guide clinicians, parents and stakeholders: *people in general do not make choices by acting only as rational balancers of risk (safety) and reward (efficacy)* [42]. These drivers, patterns and irrational practices may also be different due to the specific characteristics of this vulnerable population. This is at present still a poorly explored area in medicine, even more when we focus on perinatal or neonatal medicine.

This means that progress on rational use of medicines in neonates will not only depend on the generation of high-quality data on efficacy and safety (*“knowledge”*) and the related new tools (*“methods”*) to assess these outcome variables, but also on the subsequent approaches to facilitate access to such data and the development of implementation strategies. Data access and implementation strategies should not only facilitate technical access to data or guidelines, but should also consider the most effective strategies (*“skills”*) to approach caregivers not only as rational decision makers, but also cover the psychosocial aspects involved in the decision process of medicines prescription.

In conclusion, to further improve rational medicines use, we need more data, and tools to assess efficacy and safety in neonates. Moreover, we should facilitate access to such data, and explore strategies for effective implementation. This is because prescription practices are not only rational decisions, but also have psychosocial aspects that may guide clinicians to irrational practices, in part influenced by the psychosocial characteristics of this population.

## Figures and Tables

**Table 1:** Illustrations of clinical relevant adverse drug reactions in neonates, with the mechanisms involved.

Compound	Clinical Syndrome	Mechanisms Involved	Potential Similarities
Sulfisoxazole	“kernicterus”	Highly albumin bound antibiotic, competitive with endogenous compounds, including bilirubin. This results in higher free bilirubin concentrations and subsequent kernicterus.	Similar effects can be anticipated for other high protein bound medicines such as ceftriaxone or diphantoine.
Chloramphenicol	“grey baby syndrome”	Impaired glucuronidation capacity, results in chloramphenicol accumulation and subsequent mitochondrial dysfunction, circulatory collapse and death.	Similar effects can be anticipated for other glucuronidation dependent drug metabolism compounds, such as paracetamol or propofol.
Kaletra (lopinavir/ritonavir)	“alcohols”	Kaletra syrup contains both ethanol and propylene glycol. Impaired metabolic clearance results in accumulation, and subsequent hyperosmolality, lactic acidosis, renal toxicity, central nervous system impairment, cardiac arrhythmia, hemolysis and collapse.	Explained by ethanol/propylene glycol, competition for hepatic metabolic elimination.
Codeine by breastfeeding	“SIDS”	Exposure to morphine after conversion from codeine, related to an ultrafast metabolizer maternal genotype. The newborn has a poor glucuronidation and renal elimination capacity, resulting in accumulation, sedation, and sudden infant death syndrome.	Similar effects can be anticipated by other analgesics, such as oxycodone.
Ceftriaxone + Calcium	“collaps”	Simultaneous administration of calcium containing infusions and ceftriaxone results in intravascular precipitate, as observed during autopsy.	May be similar for other “mixtures” with calcium containing formulations.
Topical iodide	“hypothyroidism”	More pronounced skin permeability and higher body surface area results in more effective absorption of iodine with subsequent suppression of thyroid function.	Similar for other topical compounds, e.g., steroids and hexachlorophene.
